# The Cost of Reproduction in a Cooperatively Breeding Mammal: Consequences of Seasonal Variation in Rainfall, Reproduction, and Reproductive Suppression

**DOI:** 10.3389/fphys.2021.780490

**Published:** 2021-11-19

**Authors:** Paul Juan Jacobs, Daniel William Hart, Tobias Suess, Andries Koch Janse van Vuuren, Nigel Charles Bennett

**Affiliations:** Department of Zoology and Entomology, Mammal Research Institute, University of Pretoria, Pretoria, South Africa

**Keywords:** oxidative stress, redox balance, mole-rat, cooperative breeder, seasonal, reproductive suppression, hormesis, oxidative shielding

## Abstract

Biological investments, such as reproduction, are influenced by both biotic and abiotic factors and their interactions. The trade-off between reproduction and survival has been well established. Seasonally breeding species, therefore, may exhibit variations in these trade-offs, but there is a dearth of knowledge concerning this. This study investigated the physiological cost of reproduction (measured through oxidative stress) across seasons in the cooperatively breeding highveld mole-rat (*Cryptomys hottentotus pretoriae*), one of the few seasonal breeding mole-rats. Oxidative stress indicates elevated reactive oxygen species (ROS) levels, which can overwhelm antioxidant defences resulting in damaged proteins, lipids and DNA, which overall can reduce longevity and compromise reproduction. Oxidative markers such as total oxidant status (TOS-measure of total peroxides present), total antioxidant capacity (TAC), oxidative stress index (OSI), and malondialdehyde (MDA) are utilised to measure oxidative stress. In this study, breeding and non-breeding male (NBM) and female mole-rats were captured during the dry season (breeding period) and wet season (non-breeding period). There was an apparent cost of reproduction in the highveld mole-rat; however, the seasonality pattern to the cost of reproduction varied between the sexes. Breeding females (BFs) had significantly higher MDA during the breeding period/dry season in comparison to the non-breeding period/wet season; this is possibly a consequence of bearing and nursing offspring. Contrastingly, breeding males (BMs) showed increased oxidative damage in the non-breeding/wet season compared to the breeding/dry season, possibly due to increased activities of protecting their mating rights for the next breeding/dry season, but this was not significant. Interestingly, during the non-breeding period/wet season, non-breeding females (NBFs) are released from their reproductive suppression, which resulted in increases in TOS and OSI, which again indicated that just the mere ability to be able to breed results in a cost (oxidative stress). Therefore we can speculate that highveld mole-rats exhibited seasonal variation in redox balance brought about by variation in abiotic variables (e.g., rainfall), physiology and behaviour. We conclude that physiological changes associated with reproduction are sufficient to induce significant acute oxidative stress in the plasma of female highveld mole-rats, which become alleviated following transition to the non-breeding season/wet period suggesting a possible hormetic effect.

## Introduction

Life history theory is defined by how an animal spreads both the costs of reproduction and survival over its lifespan, which is influenced by abiotic (physical environment) and biotic (living components in the environment) factors and their interactions (Brommer, 2000; Chainy et al., 2016; Withers et al., 2016; Varpe, 2017; Dantas et al., 2021). Abiotic factors (e.g., temperature, water availability, photoperiod, salinity) vary for animals living in habitats with demarcated seasonality, where resource availability (e.g., food) is generally dependant on rainfall or other factors (Schradin and Pillay, 2006; Bronson, 2009). Reproduction is a high investment biological process that requires considerable resources and energy (particularly from females) for the subsequent production and development of offspring (Speakman, 2008). Thus, many mammals have adopted seasonal reproductive strategies where the birth of young generally coincides with periods of higher rainfall and/or increased resource availability, ensuring maximal offspring growth rates and survival (Bronson, 1985, 1989; Skinner and Chimimba, 2005). Conversely, when environmental conditions become unfavourable, several species cease reproductive activity and channel efforts into survival (Khokhlova et al., 2000; Belhocine et al., 2007).

Besides increased resource needs, physiological costs associated with reproduction can impair the functionality of other processes leading to possible costs related to reproductive effort (Harshman and Zera, 2007; Speakman, 2008). The cost of reproduction hypothesis predicts that animals require a trade-off into high investment processes such as those between reproduction, survival and longevity (Speakman et al., 2015; Blount et al., 2016; Costantini, 2016; Alonso-Alvarez et al., 2017; Viblanc et al., 2018). Reproduction, and the subsequent investment in offspring, has been linked to compromised survival, with prolific reproduction associated with a significantly shorter life span (Williams, 1966; Brooks and Garratt, 2017; Lemaître and Gaillard, 2017). For example, in the collared flycatcher *Ficedula albicollis* repeated reproductive effort compromises clutch size in older individuals (Gustafsson and Pärt, 1990). Reactive oxygen species (ROS) may be of profound biological importance as it has been demonstrated to mediate these high investment processes (Alonso-Alvarez et al., 2004; Dowling and Simmons, 2009; Viblanc et al., 2018; Costantini, 2019). Aerobic organisms constantly produce ROS from metabolism, where excessive ROS is reduced through antioxidants, whereby the body is trying to maintain redox balance and reduce ROS to below harmful levels (Birben et al., 2012; Jacobs et al., 2020, 2021b). When ROS levels are elevated above normal levels, a state of oxidative stress (OS), can overwhelm antioxidant defences resulting in damaging proteins, lipids, and DNA (Sies, 1991; Finkel and Holbrook, 2000; Costantini, 2008), all of which can compromise both reproduction and survival. ROS is generally considered to be detrimental to cellular integrity; however, ROS have essential physiological functions such as signal transduction (Chainy et al., 2016), oxygen sensing (Acker et al., 2006), the immune system (Yang et al., 2013), inflammatory response (Giordano, 2005), osmo-protective signalling (Burg et al., 2007), regulation of gene expression (Turpaev, 2002), and cellular functions (Droge, 2002). Initially elevated ROS levels leading to OS was thought to be one of the primary costs to reproduction in the life history trade-off between survival and reproduction (Sainz et al., 2000; Alonso-Alvarez et al., 2004; Costantini, 2008; Stier et al., 2012). But recent findings widely debate an oxidative cost to reproduction, as there is uncertainty whether there is an actual oxidative cost to reproduction (Monaghan et al., 2009; Metcalfe and Monaghan, 2013; Speakman et al., 2015; Costantini, 2019).

Alternative hypotheses have been proposed to explain no cost or reduced oxidative cost to reproduction, which include the oxidative shielding hypothesis (Blount et al., 2016) and hormesis during reproduction/oxidative conditioning hormesis hypothesis (Costantini, 2014; Luna-López et al., 2014; Alonso-Alvarez et al., 2017; Oliveira et al., 2018). The oxidative shielding hypothesis came about through the observed trends in reproductive females where in certain tissues and markers, breedering population members show reduced oxidative damage when compared to their non-breedering counterparts (Schmidt et al., 2014; Blount et al., 2016; Viblanc et al., 2018). Key to this hypothesis includes the pre-emptive reductions in levels of oxidative damage in order to protect offspring and the mother by upregulating antioxidant defences (Blount et al., 2016). This pre-emptive protection is speculated to occur from oocyte maturation, gestation and lactation (Blount et al., 2016). Similarly, males may demonstrate oxidative shielding, but this requires investigation. While, the hormesis hypothesis proposes that during reproduction, ROS intermediate may trigger pathways to reduce oxidative damage, with the effectiveness of the hormetic effect based on the exposure of ROS resulting in either a U or J shaped dose response curve (Costantini, 2014; Luna-López et al., 2014; Alonso-Alvarez et al., 2017; Oliveira et al., 2018). In this case, a mild oxidative stressor will allow the animal to cope with the stressed event and may benefit from elevated ROS levels (Luna-López et al., 2014). These two hypothesis provide alternative explanations as to why individuals either demonstrate no cost or a reduced oxidative in certain markers or tissues (Nussey et al., 2009; Ołdakowski et al., 2012, 2015; Brzęk et al., 2014; Christensen et al., 2015; Blount et al., 2016; Vaanholt et al., 2016; Vitikainen et al., 2016).

As reproduction can be seasonal due to the seasonality of abiotic factors, so may be the cost of reproduction (i.e., seasonal variation in OS). Studies focussing on seasonal changes in redox balance can be affected by seasonal climatic factors such as temperature (Bhat et al., 2008; Chainy et al., 2016; Jacobs et al., 2020, 2021b) or photoperiod (Bonda-Ostaszewska et al., 2012). Some examples include rats exposed to summer temperatures possessing higher OS and low levels of antioxidant enzymes in their erythrocytes compared to the winter season (Bhat et al., 2008). Furthermore, extreme variability in climate during the summer (e.g., heat waves) also results in OS (Jacobs et al., 2020, 2021b). Additionally, increased OS resulted from low food availability in Seychelles warblers (*Acrocephalus sechellensis*) (van de Crommenacker et al., 2011). However, there is still a vast dearth of knowledge regarding how redox balance may fluctuate due to changes between breeding seasons (breeding vs. non-breeding) brought about by environmental alterations (wet vs. dry season) (Chainy et al., 2016).

Consequently, two essential questions still exist in oxidative ecology; firstly, is there an oxidative cost to reproduction? Secondly, if there is a reproductive cost, does it change across seasons? It has been recommended that species that are cooperative breeders be used to investigate the oxidative cost to reproduction since non-breeders occur naturally and not as a result of biological (poor body condition) or artificial (experimental manipulation) reasons (Costantini, 2016). Cooperative breeding is a social system where offspring remain in their natal group past maturation, do not contribute to the colony reproductive effort and gain indirect fitness benefits from group living (Bennett and Faulkes, 2000; Lukas and Clutton-Brock, 2012). For non-cooperatively breeding species, a confounding effect of non-reproduction may be due to the animals being in poor condition (high OS) and actively choosing not to reproduce (Giudici et al., 2010; Costantini, 2016). Therefore, conclusions drawn from these species would be misleading as they would suggest there is no cost to reproduction, as the non-breeding individuals in the study may be in poor health and have a higher OS (Costantini, 2016).

The subterranean family, the Bathyergidae (African mole-rats), which contains several co-operatively breeding species, has allowed for many essential revelations in mammalian evolution, behaviour, and physiology (Bennett and Faulkes, 2000; Ivy et al., 2020; McGowan et al., 2020; Barker et al., 2021; Jacobs and Oosthuizen, 2021). Social organisation in African mole-rats ranges from strictly solitary to social to even eusocial, where group size can be in excess of 300 individuals and breeding occurs either seasonally or throughout the year depending on the species (Bennett and Faulkes, 2000). Social and eusocial bathyergids exhibit cooperative breeding and a reproductive division of labour where reproduction is often monopolised by a single breeding female (BF) and one to three of the largest males [breeding males (BMs)] within the colony (Bennett and Faulkes, 2000). The remaining colony members are non-reproductive [non-breeding females (NBFs); non-breeding males (NBMs)] and are reproductively quiescent, where non-reproductive members can reproduce but are socially reproductively suppressed by the dominant breeding individuals (Bennett and Faulkes, 2000). This reproductive suppression can be physiological (Bennett et al., 2018; Medger et al., 2019; Blecher et al., 2020) or behavioural (e.g., incest avoidance) (Burda et al., 1990; Bennett et al., 1996, 1997; Lutermann et al., 2013) or even the two in unison (Bennett et al., 1996). Non-breeding colony members dispersal from their natal colonies during periods of high rainfall when the soil characteristics are optimal for excavation and digging (Jarvis et al., 1994; Molteno and Bennett, 2002; Scantlebury et al., 2006). During such times, reproductive inhibition in non-breeding colony members may be relaxed because of the greater probability of successful independent reproduction (Molteno and Bennett, 2002). This phenomenon of relaxation of suppression has been found in several social African mole-rat species (Bennett, 1989; Spinks et al., 1997, 1999; Janse van Rensburg et al., 2002).

The social highveld mole-rat (*Cryptomys hottentotus pretoriae*) experiences distinct seasonal climates with wet, warm summers (wet season: December to March) and cool, dry winters (dry season: April to November). The breeding period occurs during the dry season from April to July (seconds litters are possible in September) so that they young are weaned by the wet season when food resources are more abundant and may increase pup survival (Janse van Rensburg et al., 2002; Hart et al., 2021). Litters born in captivity show a gestation period of 63–66 days, with 1–3 pups born per litter (Malherbe et al., 2003). Males, regardless of reproductive status, show similar reproductive activation throughout the year, indicating that there is little physiological reproductive suppression in males and being the dominant breeder is what separates BMs from NBMs (Janse van Rensburg et al., 2002). Reproductive suppression is apparent in NBF highveld mole-rats during the dry/breeding season (Janse van Rensburg et al., 2002), whereas during the non-breeding/wet season, maximal dispersal of both NBMs and NBFs and relaxation of reproductive suppression in NBFs are observed (Janse van Rensburg et al., 2002; Molteno and Bennett, 2002; Young et al., 2010).

In the current study, we set out to investigate seasonal differences in the oxidative cost to reproduction in a wild population of seasonally breeding social highveld mole-rats (*C. hottentotus pretoriae*), using a non-lethal and minimally invasive (only a single blood sample and no euthanasia) method (Jacobs et al., 2021a). We predicted that seasonal differences in oxidative markers (total antioxidant capacity-TAC; total oxidant status-TOS; OSI-Oxidative stress index; MDA-malondialdehyde) would be similar between BMs and NBMs throughout the year as both BMs and NBMs show the ability to actively breed throughout the year by possessing sperm in the testes and vas deferens. Contrastingly, we predicted BFs would show higher TOS, OSI, and MDA values if they follow the oxidative cost to reproduction hypothesis and reduced or no change to TOS, OSI, and MDA if there is oxidative shielding during the breeding/dry season than the non-breeding/wet season. Hormetic effects will be analysed *a posteriori*. The NBFs are expected to possess lower TOS, OSI, and MDA values than BFs throughout the year as they do not breed or become pregnant in the confines of the colony. Lastly, we expect TAC values to be similar between NBFs and BFs.

African mole-rats, particularly the eusocial species have already been critical in investigating a redox cost of reproduction in blood plasma. Jacobs et al. (2021b) established that in the long-lived eusocial naked mole-rat (*Heterocephalus glaber*), there is an inherent cost to breeding as BFs exhibited significantly higher total oxidant status (TOS), which resulted in a higher OSI, in comparison to NBFs. At the same time the BFs of the eusocial Damaraland mole-rat (*Fukomys damarensis*) exhibited signs of oxidative shielding, where BFs resort to pre-emptive reductions in oxidative damage and/or stress during sensitive periods of reproduction (Schmidt et al., 2013, 2014; Blount et al., 2016) resulting in the BFs possessing similar TOS and OSI to NBFs (Jacobs et al., 2021b).

## Materials and Methods

### Ethics Approval

All experimental animal procedures were approved by the University of Pretoria Faculty of Veterinary Science Animal Ethics Committee under the project code of NAS 128/2020. All methods were performed in accordance with the relevant guidelines and regulations. All experimental procedures were carried out in accordance with the recommendations in the Guide for the Care and Use of Laboratory Animals of the National Institutes of Health (National Research Council, 2010).

### Reagents

Unless otherwise stated, all chemicals and reagents used in this study were obtained from Merck (Pty) Ltd. (Gauteng, South Africa).

### Animal Capture

A total of 31 and 35 wild highveld mole-rat (Table 1) were captured during the non-breeding period/wet season (namely January 2021) and breeding period/dry season (namely June 2021), respectively, at the Pretoria Botanical Gardens (25°44′13.92″ S, 28°16′24.24″ E), Gauteng, South Africa. Animals were caught using Hickman live traps baited with sweet potatoes (Hickman, 1979). Once captured, the animals were transported to the mole-rat laboratory at the Department of Zoology and Entomology (25°45′13.3″ S, 28°13′50.9″ E), University of Pretoria, Hatfield, South Africa. On capture, the body mass of each animal was recorded to the nearest 0.01 g (Scout Pro SPU123; Ohaus Corporation, Pine Brook, NJ, United States). Unfortunately, due to animals being wild-caught, age could not be determined in the individuals.

**TABLE 1:** The total number of breeding females (BFs), breeding males (BMs), non-breeding females (NBFs), and non-breeding males (NBMs), highveld mole-rats (*Cryptomys hottentotus pretoriae*) captured during the non-breeding period/wet season (January 2021) and breeding period/dry season (June 2021).

	BFs	BMs	NBMs	NBFs
Non-breeding period/wet season	7	8	6	10
Breeding period/dry season	9	9	11	6

### Determination of Reproductive Status

All individuals >40 g were used in this study as histological examination has shown these animals to be reproductively mature (Bennett and Faulkes, 2000; Hart et al., 2021). The BMs were distinguishable from NBMs by their large inguinal testes and yellow staining around the mouth (Jarvis and Bennett, 1993; Bennett and Faulkes, 2000; Hart et al., 2021). In addition, BFs possessed prominent axillary teats and a perforated vagina, which was absent in the NBFs (Burland et al., 2004).

### Animal Housing

Mole-rats were temporarily housed in plastic crates (49.5 cm × 28 cm) and provided with wood shavings and paper towelling as nesting material. Animal room temperatures ranged between 24.5 and 26°C, were maintained on a 12L:12D photoperiod, with 50–60% relative humidity. The mole-rats were fed sweet potato, gem squash, carrots and apples daily. To ensure that sampling was as accurate as possible, animals captured from the same tunnel system (thus apart of the same colony) were maintained together whilst in captivity. Animals were kept for <1 week before being returned to their site of capture.

### Blood Sampling

All blood samples were collected within 72 h of the animals being captured. Bleeding occurred between 08:00 and 13:00 as follows: the animals were handheld, and venous blood samples were collected from the hind foot. Approximately 300–500 μl of blood was collected into heparinised micro-haematocrit tubes. The blood was centrifuged at 1,300 × *g*, and the resulting plasma was decanted and stored at −80°C until further analysis (<1 month). Only 1% or less of the total body mass of the individual of blood was allowed to be collected as set out by the University of Pretoria, Faculty of Veterinary Science Animal Ethics Committee.

### Total Antioxidant Capacity Assay

Plasma TAC levels were quantified using a commercially available kit (Antioxidant Assay Kit, Cayman Chemical Co., Ann Arbor, MI, United States) which measures the oxidation of ABTS (2,29-Azino-di- [3-ethylbenzthiazoline sulphonate]) by metmyoglobin, which is inhibited by non-enzymatic antioxidants contained in the sample. Oxidised ABTS is measured by spectrophotometry at a wavelength of 750 nm. The capacity of antioxidants in the sample to inhibit oxidation of ABTS is compared with the capacity of known concentrations of Trolox, and the results are expressed as micromole Trolox equivalents per litre (μmol Trolox equivalents/L). Samples were run in duplicate and only once per plate with a repeatability of *r* = 0.94. Intra-assay variability (%CV) was 3.3%.

### Total Oxidant Status Assay

Plasma TOS levels were measured through Erel’s method (Erel, 2004). Briefly, this method is based on the oxidation of ferrous ion to ferric ion in the presence of various oxidative species. The oxidation reaction is enhanced by glycerol molecules, which are abundantly present in the reaction medium. The ferric ion makes a coloured complex with xylenol orange in an acidic medium. The colour intensity, measured spectrophotometrically is related to the total amount of oxidant molecules that are present in the sample. The results are expressed in terms of micromole hydrogen peroxide equivalent per litre (μmol H_2_O_2_ equivalent/L). Samples were run in duplicate and not repeated once per plate with a repeatability of *r* = 0.98. Intra-assay variability (%CV) was 5.9%.

### Oxidative Stress Index

Oxidative stress was determined by the TOS:TAC ratio, which represents the OSI arbitrary unit, which was calculated as follows: OSI = [(TOS, μmol H_2_O_2_ equivalent/L)/(TAC, μmol Trolox equivalent/L)] × 100 (Bitiren et al., 2010; Jacobs et al., 2021a,b).

### Malondialdehyde Lipid Peroxidation

The concentration of MDA was measured in all plasma samples collected and was quantified using a commercially available kit (Sigma-Aldrich, cat. No. MAK085, A6283, 258105, and 360465), following standard procedures (Halliwell and Chirico, 1993). Polyunsaturated fatty acids (lipids) are susceptible to oxidative attack through ROS, resulting in MDA. The kit determines MDA content by reacting with thiobarbituric acid (TBA) to form a colourimetric complex at 532 nm. Absorbance was read using Spectramax M2 plate reader (Molecular Devices Corp., Sunnyvale, CA, United States) and compared to a 2 mM MDA standard (2–10 nmol/ml). Samples were run in duplicate with repeatability of *r* = 0.9.

### Study Site Climate Analysis

We followed the climate analysis outlined by Wallace et al. (2021) to analyse seasonal differences at the study site. Climate data at the study site from 1981 to 2020 were retrieved from ERA5-Land dataset managed by the Copernicus Climate Change Service (Muñoz Sabater, 2019). Rainfall and soil moisture (at both depths) were compared separately between seasons (non-breeding period/wet: December to March; breeding period/dry: April to November) using a Wilcoxon-test. Data are presented as mean ± standard error (s.e.m).

### Statistical Analysis

All statistical analyses were performed in R 4.0.5 (R Development Core Team, 2018). Males and females were split for all analyses. The normality of the response variables (OSI, TAC, TOS or MDA) was determined using Shapiro Wilk tests (S-W). Homogeneity of all dependent variables was confirmed with Levene’s test. Log-transformation was attempted to normalise all non-normal data. Normally distributed dependent variables were analysed using a linear model, whereas non-normally distributed dependent variables were analysed by generalised linear mixed models fitted with link-log function using the *lme4* package (Bates et al., 2015). *Post hoc* comparisons were conducted using Tukey’s HSD pairwise comparisons using the *emmeans* package (Lenth et al., 2018). Each model contained OSI, TAC, TOS or MDA as the response variables and breeding status (breeding or non-breeding) and season (non-breeding period/wet season or breeding period/dry season) as predictors, with all two-way interactions (breeding status × season) included. Body mass was included as a covariant in all models. Data are presented as mean ± standard error (s.e.m) and a *p*-value of ≤0.05 was defined as significant.

## Results

### Study Site Climate

**FIGURE 1:**
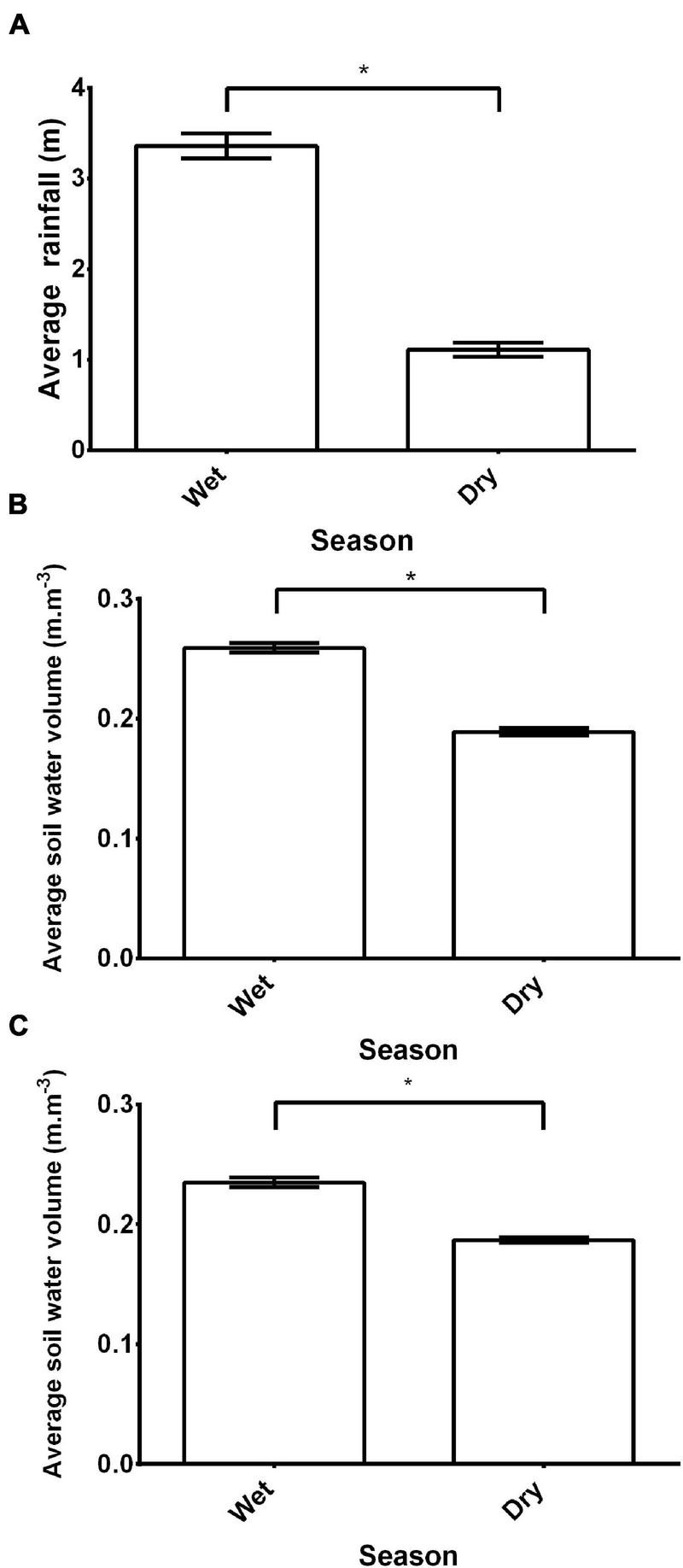
The seasonal variation [(wet: December to March) or dry: (April to November)] in **(A)** rainfall (m) and soil volumetric moisture (m.m^–3^) at **(B)** 0–7 cm and **(C)** 7–27 cm for the Pretoria Botanical Gardens (25°44′13.92″ S, 28°16′24.24″ E), Gauteng, South Africa. The climate variables was retrieved from ERA5-Land, a freely accessible dataset managed by the Copernicus Climate Change Service. Mean ± s.e.m. An asterisk (*) indicates significance (*p* ≤ 0.05).

The study site received significantly more rainfall during the non-breeding period/wet season than during the breeding period/dry season (*Z* = −13.2, *p* ≤ 0.0001, Figure 1A). Consequently, soil moisture was significantly greater in the non-breeding period/wet season than the breeding period/dry season (*Z* ≥ −11.8, *p* ≤ 0.001, for both; Figures 1B,C).

### Total Antioxidant Capacity

**FIGURE 2:**
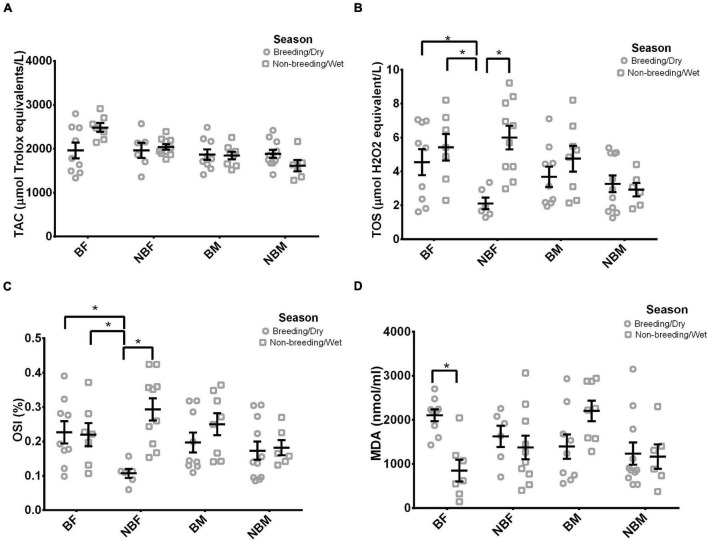
The variation of **(A)** total antioxidant capacity (TAC- μmol Trolox equivalents/L) **(B)** total oxidant status (TOS- μmol H_2_O_2_ equivalent/L) **(C)** oxidative stress index (OSI-%) and **(D)** malondialdehyde (MDA- nmol/ml) in highveld mole-rats (*Cryptomys hottentotus pretoriae*) between breeding (BF) and non-breeding (NBF) females, breeding (BM) and non-breeding males (NBM) across the non-breeding period/wet season and breeding period/dry season. Mean ± s.e.m. An asterisk (*) indicates significance (*p* ≤ 0.05).

All statistical results are presented in Table 2. Male TAC was unaffected by all primary predictors (breeding status or season), two-way interaction (breeding status × season) or body mass (Table 2 and Figure 2A). Contrastingly, female TAC levels were significantly affected by breeding status and season, respectively (Table 2). BFs (2189.4 ± 126.2 μmol Trolox equivalents/L) possessed significantly higher plasma TAC concentrations in comparison to NBFs (2011.1 ± 72.0 μmol Trolox equivalents/L). At the same time females captured in the non-breeding period/wet season (2222.4 ± 76.6 μmol Trolox equivalents/L) possessed significantly higher plasma TAC levels compared to females captured in the breeding period/dry season (1961.9 ± 123.1 μmol Trolox equivalents/L). However, breeding status × season and body mass did not significantly affect female plasma TAC levels (Table 2).

**TABLE 2:** The statistical outputs of the effects of the season (non-breeding period/wet season vs. breeding period/dry season) and breeding status (breeding vs. non-breeding), their two-way interactions and body mass on the total antioxidant capacity (TAC) of 31 female and 35 male highveld mole-rats (*Cryptomys hottentotus pretoriae*).

	Female		Male	
Variable	*t*-value	*P*-value	*t*-value	*P*-value
Season	–2.70	0.01*	0.53	0.60
Breeding status	–2.29	0.03*	–0.98	0.34
Season × breeding status	1.49	0.15	0.85	0.40
Body mass	–1.35	0.19	–1.74	0.09

An asterisk (*) indicates significance (p ≤ 0.05).

### Total Oxidant Status

All statistical results are presented in Table 3. Male plasma TOS concentrations were unaffected by all primary predictors (breeding status or season), two-way interaction (breeding status × season) or body mass (Table 3 and Figure 2B). Likewise, female TOS concentrations were unaffected by all primary predictors (breeding status or season) or body mass (Table 3), but were significantly affected by the two-way interaction (breeding status × season) (Table 3 and Figure 2B). On closer evaluation, NBFs captured in the non-breeding period/wet season possessed significantly higher plasma TOS levels in comparison to NBFs captured in the breeding period/dry season (*p* ≤ 0.0001) but possess similar TOS levels to BFs captured in both the non-breeding/wet season and breeding period/wet season (*p* ≥ 0.41, for both, Figure 1B). Contrastingly, NBFs captured in the breeding period/dry season possessed significantly lower plasma TOS concentrations in comparison to BFs captured in both the non-breeding/wet period and breeding period/wet season (*p* ≤ 0.003, for both, Figure 1B). BFs captured in the non-breeding period/wet season and breeding period/dry season possessed similar plasma TOS titre (*p* = 0.88, Figure 2B).

**TABLE 3:** The statistical outputs of the effects of the season (non-breeding period/wet season vs. breeding period/dry season) and breeding status (breeding vs. non-breeding), their two-way interactions and body mass on the total oxidative capacity (TOS) of 31 female and 35 male highveld mole-rats (*Cryptomys hottentotus pretoriae*).

	Female		Male	
Variable	*t*-value	*P*-value	*t*-value	*P*-value
Season	–0.74	0.47	–0.90	0.38
Breeding status	0.69	0.50	–1.42	0.17
Season × breeding status	–3.01	0.01*	0.68	0.50
Body mass	–1.03	0.31	–0.61	0.55

An asterisk (*) indicates significance (p ≤ 0.05).

### Oxidative Stress Index

All statistical results are presented in Table 4. Male OSI values were unaffected by all primary predictors (breeding status or season), two-way interaction (breeding status × season) or body mass (Table 4 and Figure 2C). Likewise, female OSI values were unaffected by all primary predictors (breeding status or season or body mass (Table 4) but were significantly affected by the two-way interaction (breeding status × season) (Table 4 and Figure 2C). BFs possessed similar plasma OSI values throughout the year (*p* = 0.99, Figure 2C). Non-breeding period/dry season captured NBFs possessed the lowest OSI values compared to non-breeding period/wet season NBFs and BFs of both seasons (*p* ≤ 0.03, for all, Figure 2C). BFs captured in both the non-breeding period/wet season and breeding season/dry period shared similar plasma OSI values to NBFs captured in the non-breeding period/wet season (*p* ≥ 0.43, for both, Figure 2C).

**TABLE 4:** The statistical outputs of the effects of the season (non-breeding period/wet season vs. breeding period/dry season) and breeding status (breeding vs. non-breeding), their two-way interactions and body mass on the oxidative stress index (OSI) of 31 female and 35 male highveld mole-rats (*Cryptomys hottentotus pretoriae*).

	Female		Male	
Variable	*t*-value	*P*-value	*t*-value	*P*-value
Season	0.11	0.91	–1.11	0.28
Breeding status	1.53	0.14	–1.33	0.20
Season × breeding status	–3.42	0.002*	0.62	0.54
Body mass	–0.50	0.62	0.04	0.97

An asterisk (*) indicates significance (p ≤ 0.05).

### Malondialdehyde

All statistical results are presented in Table 5. Plasma MDA concentrations in male highveld mole-rats were significantly affected by breeding status and season, respectively (Table 5), but unaffected by breeding status × season and body mass (Table 5). BMs (887.9 ± 102.4 nmol/ml) possessed significantly higher plasma MDA concentration in comparison to NBMs (605.6 ± 92.5 nmol/ml). While, males captured in the non-breeding period/dry season (880.3 ± 111.2 nmol/ml) possessed significantly higher plasma MDA titre compared to males captured in the dry/breeding seasons (653.3 ± 91.5 nmol/ml). Likewise, female highveld mole-rats plasma MDA titres were affected by season (Table 5); however, plasma MDA titre of females captured in the breeding period/dry season (957.2 ± 68.0 nmol/ml) were significantly higher than those of females captured in non-breeding period/wet season (579.6 ± 96.8 nmol/ml). However, breeding status and body mass did not affect female plasma MDA titres (Table 5). On closer investigation (breeding status × season, Table 5), BFs captured in the non-breeding period/dry season possessed significantly lower plasma MDA concentrations in comparison to BFs captured in the breeding period/dry season (*p* = 0.005, Figure 2D). Contrastingly, NBFs possess similar plasma MDA concentrations throughout the year (*p* = 0.86, Figure 2D), and NBFs possessed similar plasma MDA concentrations to BFs throughout the year (*p* ≥ 0.43, for all, Figure 2D).

**TABLE 5:** The statistical outputs of the effects of the season (non-breeding period/wet season vs. breeding period/dry season) and breeding status (breeding vs. non-breeding), their two-way interactions and body mass on the malondialdehyde (MDA) of 31 female and 35 male highveld mole-rats (*Cryptomys hottentotus pretoriae*).

	Female		Male	
Variable	*t*-value	*P*-value	*t*-value	*P*-value
Season	3.69	0.0001*	–2.24	0.03*
Breeding status	1.54	0.14	–2.59	0.02*
Season × breeding status	–2.04	0.05*	1.68	0.11
Body mass	0.53	0.60	0.67	0.51

An asterisk (*) indicates significance (p ≤ 0.05).

## Discussion

This study addressed two simple but crucial questions: Firstly, is there an oxidative cost to reproduction? Secondly, if there is a cost, is there seasonal variation to this reproductive cost controlled by a seasonal variation in rainfall? Using MDA (indicator of lipid oxidative damage) inside blood plasma as oxidative markers for whole-body oxidative status, this study highlighted a seasonality to the acute cost of reproduction in a cooperative breeding mammalian species (Argüelles et al., 2004; Veskoukis et al., 2009; Xu et al., 2014; Margaritelis et al., 2015). However, the pattern of the seasonality to the cost of reproduction varied between the sexes.

There are limitations to the current study which need to be addressed. This study involves investigating reproduction under natural conditions, which means we could not account for the oxidative state of animals before measurement, the age of individuals, or control for the potential effect of previous seasonal effects (photoperiod, temperature, soil salinity and variability in food availability). These effects in themselves can result in significant individual variation, which may explain, to some extent the spread of the data to some extent. Furthermore, the lack of antioxidant enzymatic investigation does not allow us to draw significant conclusions on the oxidative shielding hypothesis (Blount et al., 2016). Lastly, since only one damage marker was investigated, erroneous conclusions can be drawn as tissues can demonstrate variation and differential responses to oxidative stress depending on the markers used (Nussey et al., 2009; Ołdakowski et al., 2012).

Oxidative damage (MDA) in females during the breeding period/dry season in comparison to the non-breeding period/wet season strongly suggests an acute oxidative cost to reproduction is present in females, supporting the cost of reproduction hypothesis (Alonso-Alvarez et al., 2017). The oxidative cost to reproduction has previously been investigated involves reproductive status and reproductive effort. These two processes are tightly linked, as breeders may demonstrate reduced oxidative damage under normal conditions (depending on markers and tissues), however, depending on the extent of reproductive effort, oxidative stress levels can exceed normal levels and result in oxidative damage (Alonso-Alvarez et al., 2010; Garratt et al., 2010; Bergeron et al., 2011; Heiss and Schoech, 2012; Stier et al., 2012; Fletcher et al., 2013; Xu et al., 2014; Blount et al., 2016; Viblanc et al., 2018). This however is not always the case as some studies did not manipulate reproductive effort or were controlled for demonstrating when females can control their own reproductive effort, reduced or no damage may occur.

The present study used plasma markers of damage, where plasma markers have demonstrated mixed results, generally in favour of increases in oxidative damage (Alonso-Alvarez et al., 2010; Bergeron et al., 2011; Stier et al., 2012; Fletcher et al., 2013; Sharick et al., 2015), but see Nussey et al. (2009), Blount et al. (2016), Vitikainen et al. (2016). In their meta-analyses, Blount et al. (2016) demonstrated that increased reproductive effort generally results in increased oxidative damage in plasma markers. It is suggested that plasma markers demonstrate oxidative damage over tissues as it has a higher turnover and represents a more acute response to oxidative stress, but may not represent long term damage (Ołdakowski et al., 2012; Xu et al., 2014). Our data support the notion that plasma markers increase oxidative damage similar to general trend.

Oxidative shielding requires upregulation of antioxidant defences and reduced oxidative damage from oocyte maturation to lactation (Blount et al., 2016). The current study did not investigate enzymatic antioxidants or how oxidative damage in tissues may have varied, and therefor definitive conclusions about oxidative shielding and hormesis cannot be made. Despite this, these hypotheses will be used to interpret our data of plasma markers to see how well they fit. For females, plasma markers of oxidative damage did not follow the oxidative shielding hypothesis, as the damage was greatest during the peak reproductive period where shielding should be evident (Blount et al., 2016). Females may have demonstrated hormetic effects in response to oxidative stress, which is supported by two findings. Firstly, BFs demonstrated similar redox balance (OSI) between seasons suggesting that BFs were similarly stressed while breeding and not breeding implying that elevated plasma MDA levels may have been beneficial for the reproductive process congruent with the hormesis hypothesis. Secondly, the females demonstrated lower oxidative damage during the non-breeding season which suggests improved antioxidant response and repair mechanisms following the oxidative insult (Costantini, 2014; Luna-López et al., 2014). Males did demonstrate reduced oxidative damage during the peak time of the breeding season when females are receptive to mating promoting the possibility of oxidative shielding in males (Blount et al., 2016). Males did not demonstrate hormetic effects as the damage was elevated during periods when reproductive inactivity, suggesting other ecological, behavioural and seasonal effects. In light of the three hypotheses, our data supports hormesis as a potential process during the cost of reproduction in plasma markers in BFs. Oxidative stress life history theory suggests that increased ROS will occur as a result of the high energy requirements of BFs (e.g., lactation) resulting in OS and damage to macromolecules, such as lipids (Clutton-Brock et al., 1989; Monaghan et al., 2009; Metcalfe and Monaghan, 2013; Speakman and Garratt, 2014; Speakman et al., 2015). The physiological differences between BFs and NBFs during the breeding period/dry season, is the production of corpora lutea, with increased ovarian mass and volume and the processes involved in reproduction (gestation and lactation) (Janse van Rensburg et al., 2002). Corpora lutea plays a critical role in the oestrous cycle, with the primary function of progesterone secretion for reproductive functions such as implantation of blastocysts (Tomac et al., 2011). During the breeding period/dry season, particularly when BFs are pregnant, circulating progesterone and oestrogen is significantly higher (Janse van Rensburg et al., 2002). Progesterone can act as a pro-oxidant as it causes increases in ROS and nitric oxide formation (Itagaki et al., 2005; Miller et al., 2007; Yuan et al., 2016), whereas oestrogen is known for its antioxidant activity, where, when oestrogen and progesterone are present in the same concentrations, do not result in oxidative stress (Huh et al., 1994; Kireev et al., 2007). However, for BF highveld mole-rats, progesterone concentration exceeds oestradiol concentrations throughout most breeding months except during April and May (Janse van Rensburg et al., 2002). The physiological costs involved in reproduction, and progesterone secretion may explain in part an oxidative cost to reproduction, at least in plasma.

Non-breeding females has a minor increase in MDA compared to the changes observed in BFs. As a helper in cooperatively breeding groups, as are NBF highveld mole-rats, a cost to helping is incurred (Heinsohn and Legge, 1999), which could increase ROS levels. Comparatively, the cost incurred by BFs directly investing into the development of offspring would likely incur higher MDA and TOS concentrations compared to NBFs during the breeding period/dry season. Furthermore, NBFs, during the breeding period/dry season, possess almost no detectable levels of circulating progesterone or oestradiol, as a result of being reproductively suppressed, which may reduce ROS levels, and in turn reduce oxidative stress, lowering oxidative damage in comparison to BFs during this season (Janse van Rensburg et al., 2002). This is supported by significantly lower TOS and OSI by NBF compared to BFs during the breeding period/dry season. However, during the non-breeding period/wet season, there is relaxation of reproductive suppression brought about by the increased dispersal opportunities due to the increased workability of the moist soil (Janse van Rensburg et al., 2002). This relaxation of reproductive suppression allows for the return of normal reproductive function, but with no evidence of ovulation, resulting in circulating progesterone levels becoming similar to that of BFs during the non-breeding period/wet season (Janse van Rensburg et al., 2002). The consequences of relaxation of reproductive suppression [i.e., hormonal changes and increased sensitivity to LH hormone (van der Walt et al., 2001; Janse van Rensburg et al., 2002)] between the non-breeding period/wet season and breeding period/dry season of NBFs may be one of the significant contributors to the dramatic increase in TOS, and consequently, OSI of NBFs between the seasons.

Interestingly, TOS concentrations and OSI were similar between BFs in the non-breeding period/wet season and breeding period/dry season and BFs and NBFs during the breeding period/dry season, suggesting possible differences in enzymatic activity antioxidant activity between BFs and NBFs. However, definitive conclusions cannot be drawn as enzymatic antioxidants were not measured. Furthermore, although our study did not investigate antioxidant enzymes, non-enzymatic antioxidants did not increase during the breeding period/dry season, but were elevated during the non-breeding period/wet season; this was particularly evident, but not significant in BFs. Again, this could have been the result of offspring being weaned and/or ceasing physiological investment into offspring (i.e., no pregnancy or nursing) during the non-breeding period/wet season, which led to a decrease in ROS production.

Few studies have investigated whether there is an oxidative cost to male reproduction (Alonso-Alvarez et al., 2004; Garratt et al., 2012; Heiss and Schoech, 2012; Sharick et al., 2015; Romero-Haro et al., 2016). The majority of studies have found some form of oxidative cost associated with the reproductive effort in males (Alonso-Alvarez et al., 2004; Heiss and Schoech, 2012; Romero-Haro et al., 2016). For example, in the Florida scrub jay (*Aphelocoma coerulescens*), high oxidative damage levels resulted in reduced oxidative effort (Heiss and Schoech, 2012). In the zebra finch (*Taeniopygia guttata*), increased breeding effort (increased brood size) resulted in lower antioxidant defences (Alonso-Alvarez et al., 2004). NBM highveld mole-rats are reproductively suppressed only through incest avoidance and aggressive dominance interactions and as such, what separates a highveld mole-rat BM from an NBM is body size and minor testosterone changes regardless of season (Janse van Rensburg et al., 2003). It is unlikely that these changes alone are sufficient to result in significant oxidative changes, as seen by similar TOS concentrations. Interestingly, a significant discrepancy was observed in oxidative damage between the non-breeding/wet and breeding/dry season for BMs; this was in the opposite direction to what was observed in BFs. Non-significance was observed within males regardless of season and status, and may be attributed to the volatility of colony stability for males, particularly during the non-breeding period/wet season when competition is likely to be at its peak (Busch et al., 2000). Colony stability is volatile during this time due to dispersal occurring and due to the relaxation of suppression (Spinks et al., 2000; Janse van Rensburg et al., 2003). Dispersal can increase the chance of unrelated males entering the colony (Janse van Rensburg et al., 2002, 2003). During this case, incest avoidance is not sufficient to prevent mating with receptive females, and thus aggressive dominance interactions may be necessary to prevent breeding between unrelated NBMs and BFs or NBFs (Janse van Rensburg et al., 2003). Some NBMs will compete throughout the year resulting in BMs protecting their rights to breed (Janse van Rensburg et al., 2003). In contrast, some NBMs will not compete, resulting in a more stable colony structure, resulting in already established BMs not putting in extra effort to maintain breeding rights (Janse van Rensburg et al., 2003). Our findings of increased reproductive effort of BMs resulting in a compromised oxidative status (generally increased oxidative damage) is congruent in several other studies (Alonso-Alvarez et al., 2004; Heiss and Schoech, 2012; Romero-Haro et al., 2016). The added stress to outcompete other males prior to the breeding season may have an associated oxidative costs that are not significant. In conclusion, reproductive males show indications of oxidative stress not being physiologically related to reproduction, but when reproduction does occur it may demonstrate oxidative shielding to protect offspring.

This study demonstrated that there is an acute oxidative cost to reproduction at least for females. Females may also demonstrate hormesis as redox balance did not vary between breeding seasons but oxidative damage did. Males may have demonstrated oxidative shielding by keeping oxidative damage low in order to protect offspring. Interestingly, MDA plasma levels were elevated in BFs compared to NBFs but this was not significant. Unlike the naked and Damaraland mole-rat, who breed throughout the year, the highveld mole-rats exhibit distinct seasonal variation in oxidative stress (particularly oxidative damage) possibly brought about by seasonal variability across seasons and physiological changes. Furthermore, using a cooperative breeder that exhibits reproductive division of labour, we can conclude that hormonal changes and investment in reproductive processes such as corpora lutea development, investment into follicles (volume and mass) can be a significant driving force behind the oxidative cost of reproduction in plasma markers. We conclude that for females, hormonal changes associated with reproduction and seasonal effects can play a significant role in the acute oxidative stress of females. In contrast, seasonal effects and behaviour may play a significant role in oxidative stress with possible inclinations of oxidative shielding. Lastly, this study demonstrates that it is possible to determine oxidative markers in individuals without terminal endpoints and requires minimal plasma to analyse oxidative markers. Therefore, future studies should consider gathering longitudinal data to determine how oxidative markers may change over multiple seasons within the same individuals in seasonally breeding species using minimally invasive techniques. Additionally, we urge that in future research, oxidative markers should be measured both during the dry/wet or breeding/non-breeding periods to determine whether a reproductive cost might be apparent or not in different species.

## Data Availability Statement

The original contributions presented in the study are included in the article/supplementary material, further inquiries can be directed to the corresponding author.

## Ethics Statement

The animal study was reviewed and approved by the University of Pretoria Faculty of Veterinary Science Animal Ethics Committee under the project code of NAS 128/2020.

## Author Contributions

PJ, DH, TS, and AJ contributed to the conception, design and sample collection of the study. PJ performed the laboratory analysis. PJ and DH performed the statistical analysis and wrote the first draft of the manuscript. NB did the funding acquisition and supervision. All authors contributed to manuscript revision, read, and approved the submitted version.

## Conflict of Interest

The authors declare that the research was conducted in the absence of any commercial or financial relationships that could be construed as a potential conflict of interest.

## Publisher’s Note

All claims expressed in this article are solely those of the authors and do not necessarily represent those of their affiliated organizations, or those of the publisher, the editors and the reviewers. Any product that may be evaluated in this article, or claim that may be made by its manufacturer, is not guaranteed or endorsed by the publisher.
